# Short-term escitalopram treatment and hippocampal volume

**DOI:** 10.1007/s00213-014-3771-3

**Published:** 2014-10-18

**Authors:** Beata R. Godlewska, Helge W. W. Hasselmann, Artemis Igoumenou, Ray Norbury, Philip J. Cowen

**Affiliations:** 1 Psychopharmacology Research Unit (PPRU), University Department of Psychiatry, University of Oxford, Oxford, OX3 7JX UK; 2 Violence Prevention Research Unit (VPRU), Barts and The London School of Medicine and Dentistry, Queen Mary University, London, EC1M 6BQ UK; 3 Department of Psychology, University of Roehampton, London, SW15 5PU UK

Major depression has been associated with volume loss in hippocampal regions in most but not all meta-analyses (Arnone et al. 2012: Bora et al. 2012). In animal studies, repeated antidepressant treatment increases hippocampal neurogenesis (Malberg et al. 2000) but there have been few longitudinal studies in depressed patients assessing the effects of antidepressant medication on hippocampal volume. Recently, however, Arnone and colleagues (2013) reported that 8-week treatment with the selective serotonin re-uptake inhibitor (SSRI) citalopram in 32 depressed patients produced a bilateral increase in hippocampal volume. The aim of the present study was to see if a similar effect could be demonstrated during short-term treatment with the SSRI, escitalopram.

We studied 33 unmedicated participants (mean age 29.9, range 20–61 years, 19 female) who were diagnosed using the Structured Interview for DSM-IV as having major depression. Participants were scanned before and during (mean duration 46 days, range 38–66 days) treatment with escitalopram, 20 mg daily. Twenty of the patients were antidepressant naïve. In the remainder, the mean drug-free interval was 117 weeks (range 8–468 weeks). Clinical assessment included Beck Depression Inventory (BDI) and Hamilton Depression Rating Scale (HAM-D). Clinical response was classified as ≥50 % symptom reduction from baseline on HAM-D. All participants gave informed consent to the study which was approved by the local ethics committee.

Participants were scanned at the University of Oxford Centre for Clinical Magnetic Resonance Research on a 3 Tesla Siemens Trio Scanner with a 12-channel head coil (Siemens, Germany) and 1-mm^3^ voxel dimension (MPRAGE: repetition time = 2040 ms, echo time = 4.68 ms, flip angle = 8°, field of view = 256 mm). All FMRIB Software Library (FSL) analyses followed default settings described at http://fsl.fmrib.ox.ac.uk/fsl/fslwiki/FSL. FSL-VBM smoothing was performed with an isotropic Gaussian kernel of 2 mm. Hippocampi were automatically segmented using FSL-FIRST (Patenaude et al. 2011). A general linear model with 5000 permutations and threshold-free cluster enhancement was performed to assess voxel- and vertex-wise differences. FSL-FAST was used to estimate intracranial volume (ICV) by summing grey matter, white matter and cerebrospinal fluid volume and to normalise hippocampal volumes (hippocampal grey matter mm^3^/ICV cm^3^). As VBM may be less sensitive to subcortical structures, we additionally conducted vertex analysis, using the FIRST tool in FSL, which is specially designed to analyse shape and volume differences in subcortical regions (Patenaude et al. 2011). Statistical analyses were performed using SPSS 21 software (IBM, USA) with *t* tests and a two-tailed *α* = .05.

Of the 33 participants, 20 (61 %) responded during escitalopram treatment (with 16 remitters following HAM-D ≤7 criterion of remission). Overall, participants showed no change in hippocampal volume measures after escitalopram. Examining the treatment responders separately showed a trend towards reduced normalised hippocampal volume that was not explained by differences in ICV (Table 1). There was no significant correlation between change in normalised hippocampal volume and duration of escitalopram treatment (all *p* values >.05). However, the small number of subjects and restricted duration of treatment means that this lack of correlation should be treated with caution.

**Table 1:** Mean (±standard deviation) depression scores, intracranial volume (ICV, cm^3^) and hippocampal volume (mm^3^) before and during escitalopram treatment

	Pre-treatment	On treatment^a^	*t*	*p*
Entire sample (*n* = 33)				
BDI	31.7 ± 7.2	15.2 ± 11.4	7.007	<.001
HAM-D	23.0 ± 4.6	9.5 ± 7.8	9.118	<.001
ICV	1478.6 ± 158.6	1482.5 ± 129.7	−0.200	.842
Left hippocampus				
Absolute	3887.06 ± 553.09	3897.09 ± 545.42	−0.151	.881
Normalised	2.64 ± 0.36	2.64 ± 0.37	0.071	.944
Right hippocampus				
Absolute	3874.94 ± 485.70	3869.55 ± 462.71	0.130	.897
Normalised	2.63 ± 0.27	2.62 ± 0.30	0.234	.816
Responders (*n* = 20)				
BDI	31.4 ± 6.4	9.3 ± 7.5	10.157	<.001
HAM-D	23.1 ± 5.1	4.4 ± 3.9	13.679	<.001
ICV	1450.6 ± 125.0	1483.2 ± 128.1	−1.509	.148
Left hippocampus				
Absolute	3910.13 ± 661.48	3819.95 ± 604.22	0.811	.374
Normalised	2.70 ± 0.41	2.59 ± 0.41	1.638	.102
Right hippocampus				
Absolute	3837.22 ± 526.87	3784.96 ± 538.83	0.742	.313
Normalised	2.65 ± 0.32	2.56 ± 0.37	1.802	.057

^a^Mean treatment duration 46 days, range 38–66 days

In conclusion, we report no change in hippocampal volume after short-term escitalopram treatment. While the demographic and clinical characteristics of our patient sample are comparable to those reported by Arnone et al.(2013), the discrepancy between our findings could be explained by the average shorter duration of antidepressant treatment and fixed escitalopram dose we employed in our study. Previous exposure to antidepressant drug treatment might of course be important; however, 20 of our patients were antidepressant-naïve and examining these participants separately also showed no tendency to an increase in hippocampal volume. In addition, we do not have a measure of treatment concordance and it is possible that some of our patients did not take their medication. Also, it cannot be excluded that methodology contributed to differences between our results and Arnone and colleagues; though we conducted a number of different analyses, none of which showed a trend to an increase in hippocampal volume after escitalopram treatment.

Escitalopram and citalopram are closely related SSRIs, with escitalopram being the active isomer of citalopram. Some meta-analyses have reported superior efficacy of escitalopram relative to citalopram (Cipriani et al. 2009). However, there is no reason to think that their effects on brain morphology would be different.

As noted above, there are relatively few longitudinal studies of the effects of antidepressant treatment on hippocampal volume and more are needed. However, while our findings are negative, they are in broad agreement with an earlier longitudinal investigation that reported no volumetric change in hippocampus after 7 months of successful SSRI treatment in 22 depressed patients (Vythilingam et al. 2004).
